# Sphingosine 1-Phosphate Modulates Antigen Capture by Murine Langerhans Cells via the S1P_2_ Receptor Subtype

**DOI:** 10.1371/journal.pone.0049427

**Published:** 2012-11-08

**Authors:** Lukasz Japtok, Katrin Schaper, Wolfgang Bäumer, Heinfried H. Radeke, Se Kyoo Jeong, Burkhard Kleuser

**Affiliations:** 1 Faculty of Mathematics and Natural Science, Institute of Nutritional Science, Department of Toxicology, University of Potsdam, Potsdam, Germany; 2 Department of Pharmacology, Toxicology and Pharmacy, University of Veterinary Medicine Hannover, Hannover, Germany; 3 Pharmazentrum Frankfurt, Clinic of the Johann Wolfgang Goethe-University, Frankfurt/Main, Germany; 4 Neopharm Co., Ltd., Daejeon, South-Korea; Institut National de la Santé et de la Recherche Médicale U 872, France

## Abstract

Dendritic cells (DCs) play a pivotal role in the development of cutaneous contact hypersensitivity (CHS) and atopic dermatitis as they capture and process antigen and present it to T lymphocytes in the lymphoid organs. Recently, it has been indicated that a topical application of the sphingolipid sphingosine 1-phosphate (S1P) prevents the inflammatory response in CHS, but the molecular mechanism is not fully elucidated. Here we indicate that treatment of mice with S1P is connected with an impaired antigen uptake by Langerhans cells (LCs), the initial step of CHS. Most of the known actions of S1P are mediated by a family of five specific G protein-coupled receptors. Our results indicate that S1P inhibits macropinocytosis of the murine LC line XS52 via S1P_2_ receptor stimulation followed by a reduced phosphatidylinositol 3-kinase (PI3K) activity. As down-regulation of S1P_2_ not only diminished S1P-mediated action but also enhanced the basal activity of LCs on antigen capture, an autocrine action of S1P has been assumed. Actually, S1P is continuously produced by LCs and secreted via the ATP binding cassette transporter ABCC1 to the extracellular environment. Consequently, inhibition of ABCC1, which decreased extracellular S1P levels, markedly increased the antigen uptake by LCs. Moreover, stimulation of sphingosine kinase activity, the crucial enzyme for S1P formation, is connected not only with enhanced S1P levels but also with diminished antigen capture. These results indicate that S1P is essential in LC homeostasis and influences skin immunity. This is of importance as previous reports suggested an alteration of S1P levels in atopic skin lesions.

## Introduction

A multiplicity of specialized antigen presenting cells (APCs) is located in the skin, which belongs to the family of classical dendritic cells (DCs). The most important DC subset in the skin are the Langerhans cells (LCs), which recognize and capture haptens due to their pronounced endocytotic capacity [1]–[3]. In response to the detection of antigen, that have penetrated the stratum corneum, LCs migrate to skin-associated lymph nodes and cross-communicate with T-lymphocytes. They present peptide–MHC complexes, which lead to a selection of antigen-specific lymphocytes. This process is connected with the terminal differentiation of LCs and the expansion and differentiation of T-cells. Due to their prominent role in initiation of immune responses, it is not astonishing that LCs have been discussed as central participants in the development of allergic contact dermatitis (ACD) [1], [4]. However, although it is well established that LCs are prototypic APCs, their specific role in immunogenic and tolerogenic responses is still not fully elucidated [5].

It has been indicated that sphingosine 1-phosphate (S1P) plays a pivotal function in a variety of cells including immune cells [6], [7]. Thus, it is well established that the egress of T- and B-cells from lymphoid organs and their positioning in these organs are mediated by S1P signaling [8]–[11]. Moreover, S1P is involved in the modulation of several functions of natural killer cells, neutrophils, mast cells, macrophages and DCs [12]–[16]. S1P is produced from sphingosine by sphingosine kinases (SphK) from which two subtypes have been described, denoted as SphK1 and SphK2 [17], [18]. The complexity of S1P-mediated actions can be explained by the fact that it functions not only inside the cell but also acts as a ligand of G protein-coupled receptors (GPCRs), when it is secreted into the extracellular environment. Although the mechanism of release of S1P from cells is not completely understood, recent studies have drawn attention to the involvement of the ATP binding cassette (ABC) family of transporters [19], [20]. Until now five high-affinity receptors for S1P, designated as S1P_1_–S1P_5_, have been identified. The importance of these GPCRs in physiological and pathophysiological conditions has been clearly demonstrated by gene deletion studies and reverse pharmacology [21], [22].

Most recently, it has been shown that S1P influences LC homeostasis. Contact hypersensitivity (CHS) is one of the most intensively studied animal model to examine immunological mechanisms of ACD and to investigate the role of immunomodulators in this disease [1], [23]. In this model, topically administered S1P inhibited the inflammatory reaction in the sensitization as well as in the elicitation phase of CHS [24]. S1P reduced the weight and cell count of the draining auricular lymph node, as well as immigrated LCs provoked by repetitive topical administration of the hapten. It has been suggested that S1P inhibits LC migration from the side of antigen exposure to the draining lymph node via the S1P_1_ receptor subtype [24], [25]. Thus, it was presumed that a high topical administration of S1P induces a receptor internalization of S1P_1_ resulting in an unresponsiveness of LC migration. These data supply evidence that the strategy of targeting the migratory response of LCs with locally acting S1P represents an emerging option in the treatment of ACD. It should be mentioned that established immunosuppressive drugs like tacrolimus and rapamycin possess a similar action on the migratory response of skin DCs [26]. Besides this, the antigen uptake as initial step of ACD is diminished in the presence of rapamycin [27], [28]. Thus, it was of great interest to investigate whether S1P also influences antigen uptake by LCs. Indeed, here we provide evidence that S1P inhibits the ability of LCs to capture antigen. Moreover, examination of the molecular mechanism indicated the importance of the S1P_2_ receptor subtype in this key step of ACD. These data might contribute to the understanding of the biological basis for effects of S1P on LCs, and thus may help to develop novel options for the treatment of ACD.

## Materials and Methods

### Ethics Statements

All animal work have been conducted according to relevant national and international guidelines. All experiments were approved by the Bezirksregierung Hannover, Germany (Az. 33.12-42502-04-10/0074).

### Materials

S1P was synthesized as described recently [29]. S1P was dissolved in methanol and stored at −80°C. For each experiment, stored S1P was dried and freshly diluted in 0.5% fatty acid free BSA/PBS. Iscove’s modified Dulbecco’s medium (IMDM) with GlutaMax was obtained from Invitrogen (Karlsruhe, Germany). RPMI-1640, FCS superior, penicillin and streptomycin were from Biochrom (Berlin, Germany). Recombinant mouse (rm)-GM-CSF was purchased from Miltenyi Biotec (Bergisch Gladbach, Germany). Probenecid and MK571 were purchased from Biomol (Hamburg, Germany). Fatty acid free BSA, Ipegal, sodium desoxycholate, SDS, phenylmethylsulfonyl fluoride (PMSF), leupeptin, aprotinin, pepstatin, EDTA, sodium fluoride, sodium orthovanadate, FITC-labeled dextran (average mol wt 40,000), Mannan, Rottlerin, LY294002, Fumitremorgin C and siRNA were obtained from Sigma-Aldrich (Schnelldorf, Germany). Monoclonal rabbit anti-phospho Akt (Ser473) antibody (Ab), monoclonal rabbit anti-total Akt Ab, secondary anti-rabbit IgG HRP linked Ab, SDS sample buffer, dithiothreitol, LumiGlo® reagent and peroxide were purchased from New England Biolabs (Ipswich, USA). SEW2871 and FTY720-phosphate (FTY720-P) were obtained from Cayman Chemical (Michigan, USA). VPC24191 was purchased from Avanti Polar Lipids (Alabaster, USA). Reversin121 was purchased from Santa Cruz Biotechnology (Heidelberg, Germany). K6PC-5 was a generous gift from NeoPharm (Daejeon, Korea). The primary anti-mouse MHC II Ab (I-A/I-E, rat IgG2b) was purchased from Pharmingen (Hamburg, Germany). Biotinylated secondary rabbit anti-rat IgG and CyTM3-conjugated streptavidin were obtained from Jackson Immunoresearch Laboratories (West Grove, USA). eFluor®450 anti-mouse CD11c Ab and APC anti-mouse MHC class II Ab (I-A/I-E, rat IgG2b) were obtained from eBioscience (Frankfurt a. M., Germany). Primers were synthesized by eurofins MWG Operon (Ebersberg, Germany).

### Mice

Female mice (BALB/c) were purchased from Charles River (Sulzfeld, Germany). The mice were 6 to 8 weeks old, housed in groups of 5 to 6 mice per cage with a 12 h dark/light cycle and received food and water ad libitum.

### Cell Culture

The long term immature LC-like DC line XS52, which was established from the epidermis of a newborn BALB/c mouse, was kindly provided from G. Müller (Mainz, Germany). The generation of XS52 cells has been well described [30]. XS52 cells were cultured in IMDM with GlutaMax supplemented with 10% FCS superior, penicillin/streptomycin (100 IU/ml/100 µg/ml), 50 U/ml rm-GM-CSF and NS47 fibroblast supernatant (10%) in a humidified 5% CO_2_ incubator. The NS47 fibroblastic stromal cell line was kindly provided from A. Takashima (University of Toledo, OH). The generation and functional properties of NS47 cells have been well characterized [31]. Cells were cultured in IMDM with GlutaMax supplemented with 10% FCS superior, penicillin/streptomycin (100 IU/ml/100 µg/ml) in a humidified 5% CO_2_ incubator. After reaching a confluence grade of 90% the supernatants were collected and used as a supplement for XS52 culture medium.

Bone marrow-derived DCs (BM-DCs) were generated according to a standard protocol established by Lutz et al. (1999) with slight modifications [32]. Briefly, bone marrow was cultured in IMDM with GlutaMax supplemented with 10% FCS superior, penicillin/streptomycin (100 IU/ml/100 µg/ml), 20 ng/ml rm-GM-CSF. Fresh medium supplemented with rm-GM-CSF was added on days 3, 6, and 8. Analysis of the day-10 cell suspension by flow cytometry demonstrated a high yield of CD11c and MHC class II-positive cells.

### Mice Treatment and Skin Explant Culture

Mice ears were treated daily over a period of 3 days with S1P (100 µg dissolved in 80 µl methanol) or vehicle (6 mice per each group). 24 h after the last treatment mice were sacrificed by cervical dislocation. Ears were separated and divided into dorsal and ventral halves by means of two forceps. The skin explant culture was performed as recently described [33]. The dorsal ear halves were placed epidermal side up onto culture medium consisting of 250 µl RPMI-1640 supplemented with 0.4% BSA and 250 µl PBS containing FITC-labeled dextran (1 mg/ml). Incubation time with the antigen was 2 h at 37°C.

### Preparation and Evaluation of Epidermal Sheets

The preparation of epidermal sheets was described earlier [34]. In short, the epidermis was separated from the dermis by means of 3.8% ammonium thiocyanate solution. Epidermal DCs were detected with the primary anti-mouse MHC class II Ab in a 1∶150 dilution. Labelling of the Ab was visualised by using biotinylated rabbit anti-rat IgG and coupling the Ab to a fluorochrom by streptavidin-biotin technique (1∶4000, Cy^TM^3-conjugated streptavidin). The evaluation was performed using a confocal laser microscope (Leica TCS SP5, Wetzlar, Germany). After MHC class II labeled cells and FITC-labeled dextran positive cells were counted, the percentage antigen uptake was calculated. Due to insufficient MHC class II staining in one epidermal sheet/group, the epidermal sheets of 5 mice per group could be quantified.

### Flow Cytometric Analysis of Tracer Uptake

XS52 cells and BM-DCs (2 × 10^5^ cells) were rinsed twice with PBS and endocytosis was determined by adding FITC-labeled dextran solution (1 mg/ml in IMDM) containing indicated S1P receptor modulators. After the incubation period of 15 min, uptake was stopped by washing the cells four times with ice-cold CellWash® and analyzed on a FACSCanto II (BD Biosciences, Heidelberg, Germany). In some experiments, cells were preincubated with the inhibitors as described in the figure legends. BM-DCs were additionally counterstained with APC anti-mouse MHC class II Ab and eFluor®450 anti-mouse CD11c Ab. Uptake of FITC-labeled dextran was recorded in MHC class II low and CD11c positive cells (immature BM-DCs). Data shown represent uptake in experimental conditions minus background uptake (cells pulsed at 4°C).

### Western Blot Analysis

XS52 cells were seeded in six-well plates (2 × 10^5^ cells per well) and cultured for 24 h. After stimulation, cells were rinsed twice with ice-cold PBS and harvested in lysis buffer (PBS without Ca^2+^/Mg^2+^, 1% Ipegal, 0.5% sodium-desoxycholate, 0.1% SDS, 1 mM PMSF, 1 µg/ml leupeptin, 1 µg/ml aprotinin, 1 µg/ml pepstatin, 1 mM sodium orthovanadate and 50 mM sodium fluoride). Lysates were centrifuged at 14,000×*g* for 30 min. Samples containing 20–40 µg protein were boiled in SDS sample buffer (100 mM Tris/HCl, pH 6.8, 4% SDS, 0.2% bromophenol blue, 20% glycerol, 200 mM dithiothreitol) and separated by 10% SDS PAGE. Gels were blotted overnight onto PVDF membranes. After blocking with 5% non-fat dry milk for 1 h at 37°C, membranes were incubated with the appropriate primary Ab at a dilution of 1∶1000 for 2 h at room temperature, and further incubated with HRP linked secondary Ab for 1 h. Detection was performed using Lumiglo® according to the manufactureŕs protocol on ChemiDoc XRS+ (Bio-Rad Laboratories GmbH, Muenchen, Germany).

### Neon Transfection of siRNA

Down-regulation of S1P_2_ and ABCC1 was performed using the Neon Transfection system and the Neon 100 µL transfection kit (Invitrogen, Karlsruhe Germany). In detail, XS52 cells were washed with PBS and resuspended in 100 µL Neon resuspension buffer R containing siRNA (final concentration in growth medium 100 nM) for every 5×10^5^ cells. For all experiments cells were treated with S1P_2_ siRNA, ABCC1 siRNA or control siRNA consisting of a scrambled sequence that does not lead to the specific degradation of any known cellular mRNA. The cell siRNA mixture was aspirated into a 100 µL Neon Tip by the Neon Pipette. Cells were than pulsed once with a voltage of 1500 mV and a width of 30 ms in the Neon electrolytic buffer E2. After electroporation, cells were transferred into 1.9 mL of prewarmed XS52 growth medium without antibiotics supplementation. After a time period of 24 h, down-regulation of S1P_2_ receptor subtype as well as ABCC1 was confirmed by real time PCR and cells were used for experiments. The following siRNA duplexes corresponding to DNA target sequence of mouse S1P_2_ receptor or ABCC1 were used:

S1P_2_∶5′-CGA CAU UUC UGG AGG GUAA [dT] [dT]–3′, 5′-UUA CCC UCC AGA AAU GUCG [dT] [dT]-3′ and 5′-CUC UCU AUG CUA AGC ACUA [dT] [dT]-3′, 5′-UAG UGC UUA GCA UAG AGAG [dT] [dT]-3′.

ABCC1∶5′-CUC UGU UCA AGG UGU UAUA [dT] [dT]-3′, 5′-UAU AAC ACC UUG AAC AGAG [dT] [dT]-3′and 5′-GUG UAG AGU UCC GGG AUUA [dT] [dT]-3′, 5′-UAA UCC CGG AAC UCU ACAC [dT] [dT]-3′.

### Quantitative Real Time PCR

XS52 total RNA was isolated using High Pure RNA Isolation Kit (Roche Applied Sciences, Mannheim, Germany) following quality check by spectroscopy. Reverse transcriptase reaction was carried out to convert 1 µg isolated mRNA into cDNA using the FermentasAid First strand cDNA synthesis kit (Fermentas GmbH, St. Leon-Rot, Germany) according to the instructions of the manufacturer. An aliquot of cDNA solution (1 µL) was subjected to quantitative real time PCR using a LightCycler480 II and the SYBR Green PCR master mix (Roche Applied Sciences, Mannheim, Germany). GAPDH and hypoxanthine phosphoribosyltransferase 1 (HPRT1) were used as normalization controls. The thermal cycle profile used was denaturing for 10 s at 95°C, annealing primers for 10 s at 60°C, and extending the primers for 10 s at 72°C. The PCR amplification was performed at 45 cycles with monitoring fluorescence. The primer sets used were: for mouse GAPDH 5′-CCT CGT CCC GTA GAC AAA ATG-3′ (forward), 5′-TGA AGG GGT CGT TGA TGGC-3′ (revers), mouse HPRT1 5′-TGG ATA CAG GCC AGA CTT TGTT-3 (forward), 5′-CAG ATT CAA CTT GCG CTC ATC-3′ (revers), mouse S1P_2._

5′-TTA CTG GCT ATC GTG GCT CTG-3′ (forward), 5′-ATG GTG ACC GTC TTG AGCAG-3′ (revers), mouse ABCB1 5′-AGT GGA CCC AAC AGT ACT CTGAT-3′ (forward), 5′-GCA CCA ATC CCG GTG TAATA-3′ (revers), mouse ABCC1 5′-GCCC CAG TGT TAC TGG TCA-3′ (forward), 5′-CAA AAA GGTG GCG AGCAG-3′ (revers), mouse ABCG2 5′-GCC TTG GAG TAC TTT GCA TCA-3′ (forward), 5′-AAA TCC GCA GGG TTG TTGTA-3′ (revers).

### Determination of S1P by Mass Spectrometry

S1P was extracted by a modified two-step lipid extraction previously described [35]. Briefly, 1 mL medium of XS52 cells (10^6^ cells) was transferred into a glass tube and 100 pmol C17-S1P as internal standard, 100 µL of a 3N NaOH solution, 1 mL of chloroform and 1 mL of methanol/HCl (99.8∶0.2 v/v) were added. After separation, the aqueous phase was acidified with 100 µL concentrated HCl and extracted with 1.5 mL chloroform. The organic phase was evaporated and the dried lipids were resolved in 200 µL methanol. Sample analysis was performed by rapid resolution liquid chromatography/tandem mass spectrometry (LC-MS/MS) using a quadrupole/time-of flight (QTOF) 6530 mass spectrometer (Agilent Technologies, Waldbronn, Germany) operating in the positive electrospray ionization (ESI) mode. Chromatographic separations were performed by a X-Bridge column (C18, 4.6×150 mm, 3.5 µm particle size, 138 Å pore size, Waters GmbH, Eschborn, Germany). Elution was performed using a gradient consisting of eluent A (water/formic acid 100∶0.1 v/v) and eluent B (acetonitril/tetrahydrofuran/formic acid 50∶50:0.1 v/v). The precursor ions of S1P (m/z 380.2560) and C17-S1P (m/z 366.2404) were cleaved into the fragment ions of m/z 264.2700 and m/z 250.2529 respectively. Quantization was performed with Mass Hunter Software.

### Statistical Analysis

Data are expressed as the mean ± SEM of results from at least three experiments, each run in triplicate. Statistics were performed using Students t test. *P < 0.05 and **P < 0.01 indicate a statistically significant difference vs. control experiments.

## Results

### Topical Application of S1P Inhibits Endocytosis by LCs in an *in situ* Animal Model

**Figure 1:**
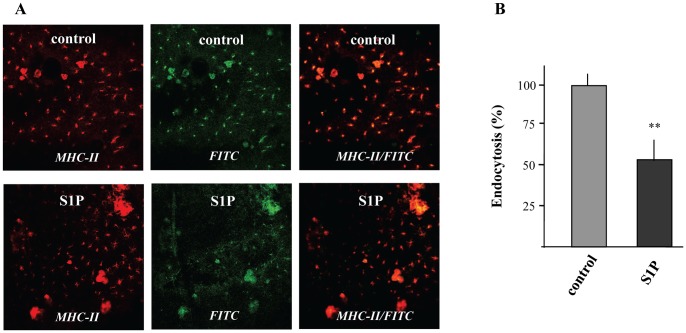
The effect of S1P on the endocytosis of FITC-labeled dextran by epidermal DCs in skin explant cultures. Mice were topically treated for 3 days with 100 µg S1P/daily. Skin explant cultures were performed and the dorsal ear halves were incubated with FITC-labeled dextran (1 mg/ml) for 2 h at 37°C. Epidermal DCs were detected via MHC class II staining and endocytosis was visualized in epidermal sheets using confocal microscopy. One out of three experiments with similar results is shown (A). The percentage of dextran uptake was calculated by counting of MHC class II- and FITC-labeled positive cells. Data are expressed as the mean ± SEM of results from at least three experiments (B). **P < 0.01 indicates a statistically significant difference vs. control experiments.

Most recently, it has been indicated that topical application of S1P attenuate inflammatory response in a murine model of ACD [24]. Nevertheless, the influence of S1P on antigen capture, which is the initial step of ACD, has not been examined. Thus, it was of great interest whether topical application of S1P also influences endocytosis by LCs. For this purpose, an *in situ* experimental approach was developed visualizing the capture of FITC-labeled dextran by epidermal DCs. Immunohistochemical analysis of epidermal sheets showed an efficient uptake of FITC-labeled dextran by LCs identified by the expression of MHC class II *in situ*. There was a significant difference on endocytosis by LCs depending whether mice were treated with vehicle or S1P. Indeed as presented in Fig. 1A, topical administration of S1P drastically reduced the endocytotic capacity of LCs. Quantitative determination revealed that uptake of FITC-labeled dextran was reduced by almost 40%, when mice were exposed to S1P (Fig. 1B).

### S1P Inhibits Endocytosis in the Murine LC-cell Line XS52 as well as in BM-DCs

**Figure 2:**
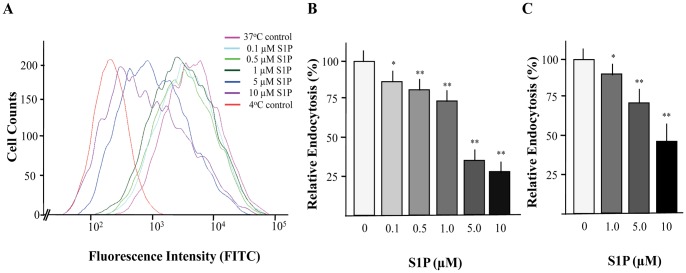
The effect of S1P on endocytosis of FITC-labeled dextran by XS52 cells and BM-DCs. Cells were incubated with FITC-labeled dextran in the presence or absence of the indicated S1P concentrations for 15 min. FITC fluorescence intensity of XS52 cells (A) and BM-DCs was analyzed by flow cytometry. Relative endocytosis by XS52 cells (B) and immature BM-DCs (MHC class II low and CD11c positive) (C) was calculated as mean values of FITC-labeled positive cells after subtraction of 4^o^C background fluorescence. Data are expressed as the mean ± SEM of results from at least three independent experiments. *P < 0.05 and **P < 0.01 indicate a statistically significant difference vs. control experiments (B,C).

The *in situ* experiments indicated that S1P influences uptake of FITC-labeled dextran. To further substantiate the role of S1P on this initial step, uptake of FITC-labeled dextran was examined on the immature LC cell line XS52. In particular, measurement of endocytosis by FACS revealed that S1P diminished the ability of XS52 cells to capture FITC-labeled dextran in a dose-dependent manner (Fig. 2A). As presented in Fig. 2B, a significant inhibition of endocytosis in response to S1P was visible at a concentration of 0.1 µM, whereas a maximal decrease of antigen capture occurred at 10 µM of S1P. Analysis of FACS data confirmed that endocytosis of FITC dextran in XS52 cells was diminished by more than 70% in the presence of 10 µM S1P. To examine whether the inhibitory effect of S1P on FITC-labeled dextran capture is restricted to XS52 cells but also visible in further DC population, BM-DCs were generated. Measurement of endocytosis clearly indicated that FITC-labeled dextran uptake by BM-DCs is also reduced in the presence of S1P in a dose-dependent manner. A maximal inhibitory effect (approximately 50%) was obvious in the presence of 10 µM S1P (Fig. 2C).

### S1P Inhibits Macropinocytosis via Modulation of Phosphatidylinositol 3-kinase (PI3K) Activity

**Figure 3:**
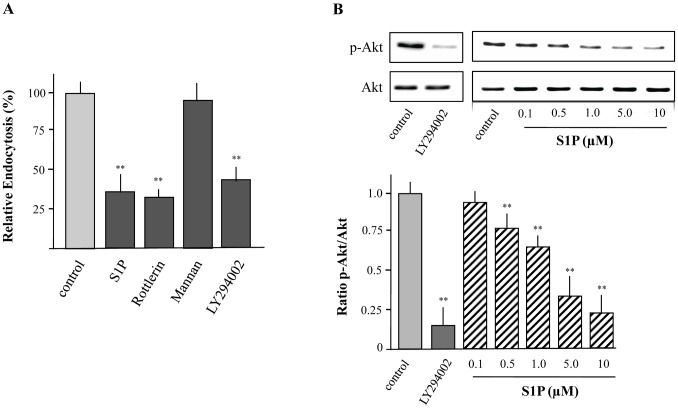
Uptake of FITC-labeled dextran by XS52 cells via macropinocytosis in a PI3K dependent manner. Cells were preincubated with Rottlerin (3 µM), Mannan (1 mg/mL), and LY294002 (10 µM) for 30 min. Then, cells were incubated with FITC-labeled dextran for 15 min. S1P (5 µM) was used as positive control. Fluorescence intensity of cells was analyzed by flow cytometry and relative endocytosis was calculated. Data are expressed as the mean ± SEM of results from at least three independent experiments. **P < 0.01 indicate a statistically significant difference vs. control experiments (A). Cells were treated with the indicated concentrations of S1P or LY294002 (10 µM) for 15 min followed by the detection of Akt activity using Western blot analysis (B). Values of the densitometric analysis are expressed as x-fold decrease of phosphorylated Akt (p-Akt) formation compared to untreated cells ± SEM from three experiments. **P<0.01 indicates a statistically significant difference versus control (B).

LCs possess at least three different mechanisms by which antigen uptake can occur, namely macropinocytosis, phagocytosis and receptor-mediated endocytosis [36]. To address potential uptake mechanisms of FITC-labeled dextran in XS52 cells, capture of this soluble endocytotic tracer was performed in the presence of Mannan and Rottlerin. When cells were pretreated with Mannan to block receptor-dependent endocytosis, FITC-labeled dextran uptake was not significantly diminished. On the contrary, preincubation with Rottlerin, which selectively inhibits macropinocytosis [37], led to a significant reduction of FITC-labeled dextran uptake indicating that macropinocytosis is the prominent mechanism how FITC-labeled dextran is captured by XS52 cells (Fig. 3A).

**Figure 4:**
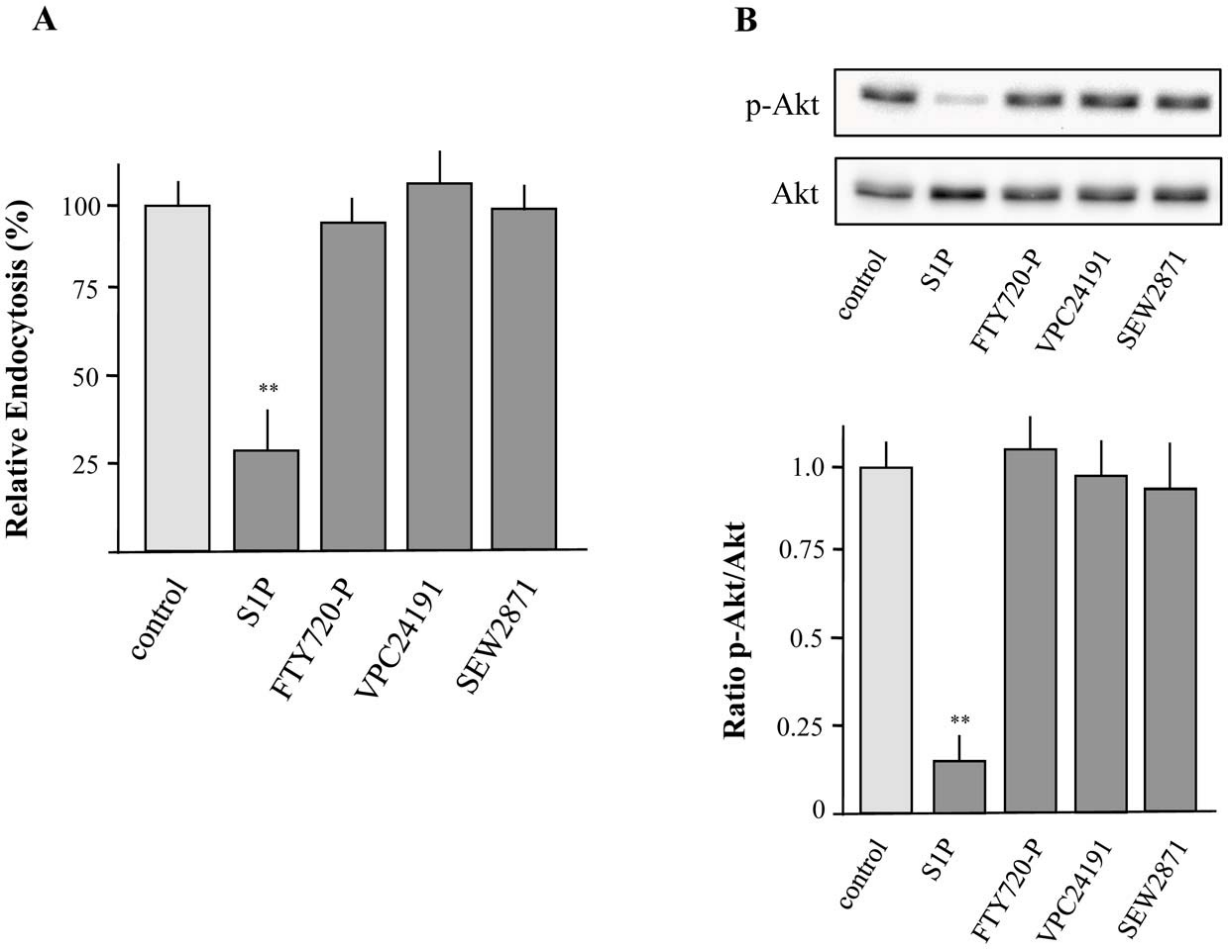
Effects of S1P receptor modulators on uptake of FITC-labeled dextran and PI3K activity. XS52 cells were incubated with FITC-labeled dextran in the presence or absence of S1P (5 µM), FTY720-P (1 µM), VPC24191 (10 µM), and SEW2871 (1 µM) for 15 min. Fluorescence intensity of cells was analyzed by flow cytometry and relative endocytosis was calculated. Data are expressed as the mean ± SEM of results from at least three independent experiments. **P < 0.01 indicate a statistically significant difference vs. control experiments (A). Cells were treated with similar concentrations of S1P, FTY720-P, VPC24191, and SEW2871 for 15 min followed by the detection of Akt activity (B). Values of the densitometric analysis are expressed as x-fold decrease of p-Akt formation compared to untreated cells ± SEM from three experiments. **P<0.01 indicates a statistically significant difference versus control (B).

**Figure 5:**
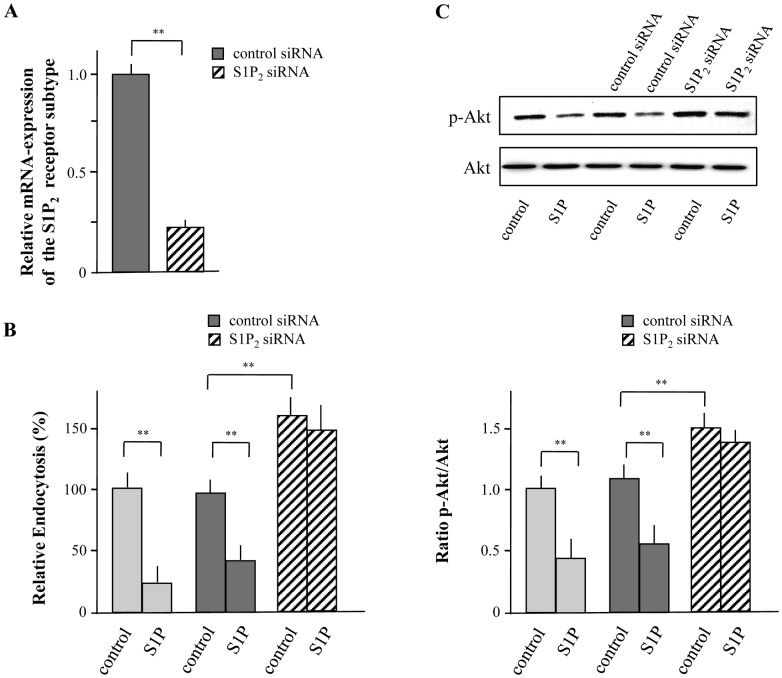
S1P inhibits macropinocytosis of FITC-labeled dextran via the S1P_2_ receptor subtype. XS52 cells were transfected with siRNA against S1P_2_ or control siRNA and silencing was detected by quantitative real time PCR (A). Transfected and control cells were incubated with FITC-labeled dextran in the presence or absence of S1P (5 µM) for 15 min and macropinocytosis was detected by flow cytometry. Relative endocytosis are expressed as the mean ± SEM of results from at least three independent experiments. **P < 0.01 indicate a statistically significant difference vs. control experiments (B). Transfected or control cells were treated with S1P (5 µM) for 15 min followed by the detection of Akt activity (C). Values of the densitometric analysis are expressed as x-fold decrease of p-Akt formation compared to untreated cells ± SEM from three experiments. **P<0.01 indicates a statistically significant difference versus control (C).

**Figure 6:**
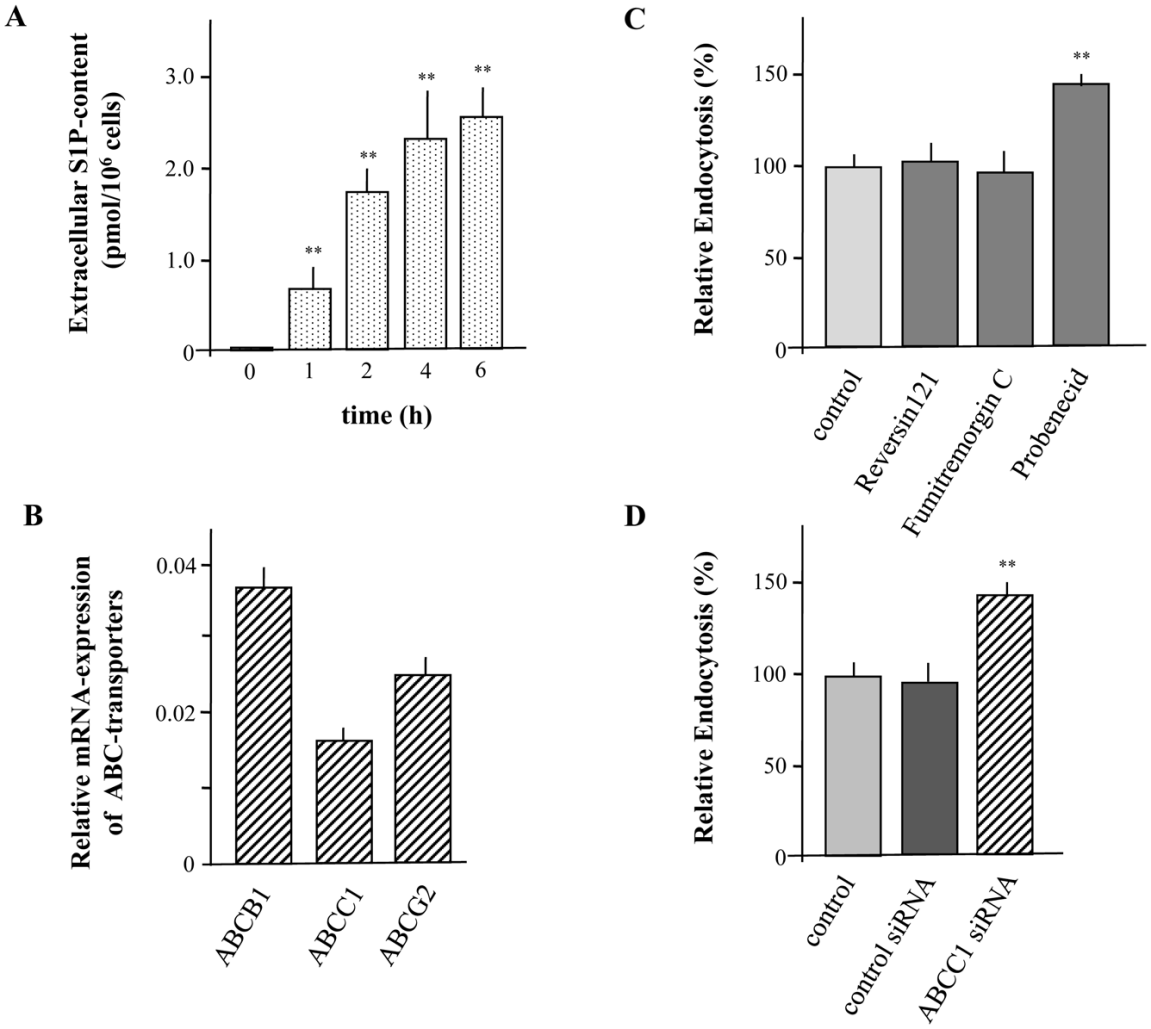
S1P is generated by XS52 cells and released to the extracellular environment. XS52 cells were cultivated over a time period of 6 h and S1P levels in the extracellular environment was detected (A). Quantitative real time PCR analysis of ABCB1, ABCC1, and ABCG2 of three different sets of cells was performed using HPRT1 and GADPH as reference genes (B). Cells were preincubated with Reversin 121 (10 µM), Fumitremorgin C (10 µM), and Probenecid (2.5 mM) for 6 h (C). Cells were transfected with siRNA against ABCC1 or control siRNA and silencing was detected by quantitative real time PCR (D). Then, cells were incubated with FITC-labeled dextran for 15 min. Fluorescence intensity of cells was analyzed by flow cytometry and relative endocytosis was calculated. Data are expressed as the mean ± SEM of results from at least three independent experiments. **P < 0.01 indicates a statistically significant difference vs. control experiments.

**Figure 7:**
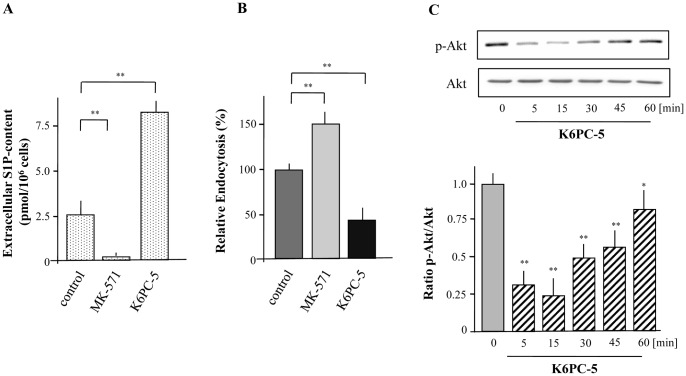
Autocrine modulation of macropinocytosis by XS52 cells via formation and release of S1P. XS52 cells were cultivated in the presence or absence of MK-571 (15 µM) and K6PC-5 (10 µM) over a time period of 6 h and S1P levels in the extracellular environment was detected (A). Cells were preincubated with MK-571 (15 µM) for 6 h and K6PC-5 (10 µM). Then, cells were incubated with FITC-labeled dextran for 15 min. Fluorescence intensity of cells was analyzed by flow cytometry and relative endocytosis was calculated. Data are expressed as the mean ± SEM of results from at least three independent experiments. **P < 0.01 indicate a statistically significant difference vs. control experiments (B). Cells were treated with 10 µM of K6PC-5 for the indicated time periods followed by the detection of Akt activity (C). Values of the densitometric analysis are expressed as x-fold decrease of p-Akt formation compared to untreated cells ± SEM from three experiments. *P < 0.05 and **P<0.01 indicate a statistically significant difference versus control.

It has been clearly demonstrated that the PI3K/Akt pathway may be involved in the regulation of antigen uptake via macropinocytosis [38]. To proof that this pathway is also essential for macropinocytosis in XS52 cells, capture of FITC-labeled dextran was performed in the presence of the PI3K-inhibitor LY294002. As expected, LY294002 prevented the phosphorylation of Akt, the main downstrean target of PI3K (Fig. 3B). This was accompanied by an inhibition of FITC-labeled dextran macropinocytosis by XS52 cells (Fig. 3A). Consequently, it was of interest to examine whether S1P-mediated inhibition of macropinocytosis is mediated via modulation of the PI3K/Akt pathway. When XS52 cells were stimulated with S1P a dose dependent reduction of Akt phosphorylation was detected (Fig. 3B). In detail, a significant inhibition of the phosphorylation status of Akt was visible with 0.5 µM of S1P, whereas a maximal reduction of phosphorylated Akt occurred with 10 µM of S1P (Fig. 3B). It should be noted that the inhibitory effect of S1P on PI3K activity is in congruence with effective concentrations to diminish macropinocytosis.

### S1P Inhibits Macropinocytosis and Phosphorylation of Akt via the S1P_2_ Receptor Subtype

It has been suggested that S1P acts as both an extracellular ligand for cell surface receptors and an intracellular signaling molecule [7]. To address whether the inhibitory effect of S1P on macropinocytosis and phosphorylation of Akt is mediated by a specific receptor subtype, the aptitude of specific S1P receptor agonists was examined. It has been shown that XS52 cells express 4 of 5 S1P, namely S1P_1–4_ but not S1P_5_ [39]. As presented in Fig 4A, neither FTY720-P, which is an agonist of all S1P receptors except the S1P_2_ receptor subtype, nor the S1P_1_/S1P_3_ agonist VPC24191 and the selective S1P_1_ agonist SEW2871 were able to impair the capacity of XS52 cells to capture FITC-labeled dextran. Congruently, phosphorylation of Akt was not modulated by the use of these agonists (Fig. 4B). These results suggest that the inhibitory effect on antigen uptake is modulated by the S1P_2_ receptor subtype or by a non-receptor mediated action. To proof this assumption, the S1P-mediated action on FITC-labeled dextran uptake and Akt-phosphorylation was determined after down-regulation of S1P_2_ by siRNA. Real-time PCR revealed that siRNA reduced the mRNA expression of S1P_2_ by more than 80% compared to control cells (Fig. 5A). Down-regulation of S1P_2_ almost completely diminished the ability of S1P to reduce macropinocytosis (Fig. 5B). In agreement S1P-mediated inhibition of Akt-phosphorylation was diminished (Fig. 5C) indicating that the S1P_2_ receptor subtype is essential for S1P-mediated modulation of macropinocytosis and inhibition of Akt-phosphorylation. Most interestingly, down-regulation of S1P_2_ drastically increased the basal endocytotic capacity of XS52 cells. Thus, the ability of XS52 cells to capture antigen was increased by more than 50% (Fig. 5B). In congruence with these results, the basal activity of phosphorylated Akt was also elevated (Fig. 5C).

### S1P Release from XS52 Cells via ABCC1 is Involved in Macropinocytosis

As down-regulation of S1P_2_ not only diminished S1P-mediated action but also affected the basal activity of XS52 cells on macropinocytosis, it can be assumed that S1P is continuously produced and secreted from XS52 cells. A significant increase of S1P into the medium could be detected over a time period of 6h (Fig. 6A). Thus, the question remains how S1P is secreted from XS52 cells. Several studies in other cell types indicate that intracellular generated S1P can be released into the extracellular environment via ABC-transporters. Especially a participation of ABCB1, ABCC1, and ABCG2 has been discussed [19]. Quantitative real-time PCR revealed that all these ABC-transporters are present in XS52 cells (Fig. 6B). Thus, macropinocytosis was examined in the presence of the ABCB1 inhibitor Reversin121, the ABCG2 inhibitor Fumitremorgin C and the ABCC family inhibitor Probenecid. In detail, Reversin121 and Fumitremorgin C did not influence FITC-labeled dextran uptake, whereas Probenecid increased the endocytotic capacity of XS52 cells by almost 50% suggesting that a member of the ABCC family is involved in S1P release (Fig. 6C). Moreover, to prove the involvement of the ABCC1, macropinocytosis was analyzed after down-regulation of ABCC1 by the use of specific siRNA as well as in the presence of the specific ABCC1 inhibitor MK-571. Real-time PCR indicated that siRNA reduced the mRNA expression of ABCC1 by almost 90% compared to control cells (data not shown). Indeed, down-regulation of ABCC1 increased the ability of LCs to capture FITC-labeled dextran by more than 40% (Fig. 6D). In agreement, FITC-labeled dextran uptake was also enhanced by the use of MK-571 confirming the participation of ABCC1 in the S1P-release (Fig. 7B). To further substantiate that ABCC1 is responsible for carrying of S1P, the content of S1P in the extracellular environment was measured in the presence of MK-571. As expected, MK-571 almost completely diminished the ability of XS52 cells to secrete S1P into the extracellular medium (Fig. 7A). To further demonstrate an autocrine mechanism of S1P on inhibition of endocytosis, S1P-formation was increased by the use of the specific Sphk1 activator K6PC-5 [40]. Stimulation of XS52 cells with K6PC-5 significantly increased extracellular levels of S1P (Fig. 7A). Consequently, it was of interest to analyse FITC-labeled dextran uptake in the presence of K6PC-5. As it is shown in Fig. 7B, macropinocytosis of FITC-labeled dextran was significantly reduced when cells were treated with the Sphk1 activator. In agreement, phosphorylation of Akt was reduced after treatment with K6PC-5 (Fig. 7C). A decrease of Akt-phosphorylation in response to the Sphk1 activator occurred already after 5 min with a maximal inhibition at 15 min.

Taken together, these data indicate that intracellular generated S1P is released via ABCC1 and acts in analogy to extracellular added S1P on the S1P_2_ receptor subtype to inhibit macropinocytosis.

## Discussion

It has been supposed that pathogens entering the body via the epidermis would encounter, be taken up, processed and finally presented in draining lymph nodes by LCs, which were therefore the initiating cells for a T cell immune response [4]. Several studies indicate that LCs possess a critical role in the development of CHS, which is a delayed-type hypersensitivity response to a topically applied hapten [41]–[44]. It has been shown that a specific depletion of LCs in the skin is connected with an inefficient transport of an antigen to draining lymph nodes, resulting in a suboptimal priming of T cells and a reduced CHS [45], [46]. Thus, it is not astonishing that several immunomodulators like cyclosporine A, tacrolimus, rapamycin, cilomilast, and glucocorticoids mediate their antiinflammatory effects via the modulation of LC functions [26], [47], [48]. Most recently, the sphingolipid S1P has also been identified to reduce the inflammatory response in the sensitization as well as the elicidation phase of CHS [24]. It has been suggested that topical administration of S1P inhibits the migration of LCs. Indeed, a S1P gradient plays a crucial role in guiding LCs from the peripheral skin site to the lymph nodes [49].

*In vitro* cell culture assays revealed that S1P is a migratory stimulus of LCs and that the S1P_1_ and S1P_3_ receptor subtypes are responsible for the migratory response [39]. Thus, inhibition of migration after a topical treatment with S1P in the CHS model can be explained by either an internalization of the S1P_1_ receptor subtype or an dysregulation of the S1P-gradient.

Nevertheless, it has also been indicated that immunomodulators like rapamycin affects not only the migratory behaviour of APCs but also their endocytotic capacity [27], [28]. In agreement, our studies of epidermal sheets demonstrated that topical treatment with S1P leads to an inhibition of antigen uptake by LCs. This is confirmed by *in vitro* cell culture assays of immature LCs showing a dose-dependent reduction of endocytosis. It is of interest that an opposite effect of S1P has been described in mature BMDCs as the endocytotic behaviour in response to S1P is increased [50], [51]. Our results indicate that endocytosis by immature BMDCs is also diminished in the presence of S1P suggesting that the modulation of antigen capture is not restricted to LCs. In this context it should be mentioned that rapid uptake of antigen by mature DCs is not as sufficient as in immature cells and moreover does not necessarily contribute to an efficient presentation of antigen on the cell surface [52].

In an immature stage LCs are able to take up antigen via several different mechanisms. As professional APCs, LCs are prone to internalize mannose via the C-type lectin receptor Langerin, as the majority of glycoproteins from bacteria and yeast are mannosylated [36]. Moreover, efficient capture of soluble antigen is mediated via macropinocytosis. LCs are a prominent cell type to pinocytose constitutively without the receipt of external stimuli, which enables LCs to screen a large volume of fluid for antigen followed by a processing and presentation of concentrated antigen [38]. Our study indicate that S1P inhibits uptake of FITC-labeled dextran via macropinocytosis, which plays a central role in the development of CHS [53]. Macropinocytosis has been characterized as an actin-dependent process that requires the Rho-family GTPases, including Rac1 and Cdc42, for actin cytoskeletal rearrangements [54]. It has been well established that this process is mediated by a modulation of the PI3K activity allowing a fine-tuned regulation of antigen uptake [38]. Noradrenaline rapidly enhances antigen capture by DCs via α(2)-adrenoceptor-mediated PI3K activation resulting in an immune enhancement following acute stress [55]. In agreement, our results indicate that S1P is able to reduce PI3K activity via an activation of the S1P_2_ receptor subtype, which consequently leads to a reduction of antigen uptake by macropinocytosis. The discrepancy between *K*_d_-values of S1P_2_ and the most effective concentration to inhibit macropinocytosis could be explained by a high unspecific binding of the S1P/BSA complex. Moreover, it has been shown that S1P in micromolar concentrations also influences PI3K activity via the S1P_2_ receptor subtype in further epidermal cells [56]. Thus, it has been shown that S1P induces cell growth arrest in keratinocytes by stimulation of S1P_2_ and subsequent reduction of PI3K activity. Most interestingly, our results indicate that down-regulation of S1P_2_ not only prevents the inhibitory effect of S1P on antigen uptake but also increase the basal level of the macropinocytotic capacity. These results provide evidence that intracellular formed S1P acts in an autocrine manner via S1P_2_ to control immunological balance of antigen capture. This hypothesis was substantiated by the use of K6PC-5 which is a direct inducer of Sphk1 [40]. This activator enhanced the extracellular level of S1P, which was accompanied by a reduced macropinocytosis. Thus, it is not astounding that reduced S1P levels have been reported in lesional skin of dogs with atopic dermatitis [57]. It seems likely that reduced S1P levels are the result of an extent metabolism via the S1P-lyase, which cleaves S1P into phosphoethanolamine and hexadecenal [58]. In this light it has clearly been highlighted that S1P-lyase activity is altered not only in atopic lesions in dogs but also in humans [59], [60].

Although our results predict the significance of S1P_2_ in the modulation of macropinocytosis, it has been pointed out that FTY720-P, which is an agonist on all S1P receptors except S1P_2_, reduced allergic inflammatory response in CHS [24]. In any case, the antiinflammatory effect of S1P should not solely be explained by a reduced antigen uptake. Several mechanisms for example a reduced migratory response of DCs, a defective T-cell stimulatory effect and a systemic effect may contribute to the protective effect of FTY720. Thus, it has been reported that topical administration of FTY720 is accompanied by systemic effects causing lymphopenia. In analogy, long-term treatment with topical administered S1P results in lymphopenia due to slightly elevated S1P-levels in plasma which could synergistically contribute to the antiinflammatory action of local S1P [24]. Moreover, it has been shown that S1P inhibits LPS-mediated IL-12p70 production in DCs. This cytokine is essential to guide differentiation of T cells into a Th1, cytotoxic/inflammatory state [61].

Yet it was not clear how S1P generated intracellularly is released from LCs reaching the S1P_2_ receptor subtype. Here, we provide evidence that the ABCC1 is involved in the secretion of the sphingolipid regulating macropinocytosis. Nevertheless, ABCC1 activity is also crucial for migration and differentiation of DCs indicating to fulfill an important physiological role in this cell type [62]. Moreover, the transporter has also been identified to secrete intracellular generated S1P from mast cells, which emphasize that ABCC1 is not an unique feature for the transport of S1P in LCs but also in further immune cells [63].

Taken together, our results indicate that the sphingolipid S1P is a tantalizing signaling molecule in LCs regulating antigen capture via the ABCC1/S1P_2_ and PI3K axis. The notion that regulatory missions of LCs are modulated directly by the S1P microenvironment contributes to the understanding of the molecular mechanism why S1P is beneficial in CHS.

## References

[1] Christensen AD, Haase C (2012) Immunological mechanisms of contact hypersensitivity in mice. APMIS 120: 1–27. 22151305 10.1111/j.1600-0463.2011.02832.x

[2] Kaplan DH (2010) In vivo function of Langerhans cells and dermal dendritic cells. Trends Immunol 31: 446–451. 21035396 10.1016/j.it.2010.08.006 PMC3002785

[3] Steinman RM (2007) Dendritic cells: understanding immunogenicity. Eur J Immunol 37 Suppl 1S53–60. 17972346 10.1002/eji.200737400

[4] Kaplan DH, Igyarto BZ, Gaspari AA (2012) Early immune events in the induction of allergic contact dermatitis. Nat Rev Immunol 12: 114–124. 22240625 10.1038/nri3150 PMC3578582

[5] Romani N, Brunner PM, Stingl G (2012) Changing views of the role of Langerhans cells. J Invest Dermatol 132: 872–881. 22217741 10.1038/jid.2011.437

[6] Japtok L, Kleuser B (2009) The role of sphingosine-1-phosphate receptor modulators in the prevention of transplant rejection and autoimmune diseases. Curr Opin Investig Drugs 10: 1183–1194. 19876786

[7] Spiegel S, Milstien S (2011) The outs and the ins of sphingosine-1-phosphate in immunity. Nat Rev Immunol 11: 403–415. 21546914 10.1038/nri2974 PMC3368251

[8] Allende ML, Dreier JL, Mandala S, Proia RL (2004) Expression of the sphingosine 1-phosphate receptor, S1P1, on T-cells controls thymic emigration. J Biol Chem 279: 15396–15401. 14732704 10.1074/jbc.M314291200

[9] Brinkmann V, Davis MD, Heise CE, Albert R, Cottens S, et al (2002) The immune modulator FTY720 targets sphingosine 1-phosphate receptors. J Biol Chem 277: 21453–21457. 11967257 10.1074/jbc.C200176200

[10] Kabashima K, Haynes NM, Xu Y, Nutt SL, Allende ML, et al (2006) Plasma cell S1P1 expression determines secondary lymphoid organ retention versus bone marrow tropism. J Exp Med 203: 2683–2690. 17101733 10.1084/jem.20061289 PMC2118149

[11] Matloubian M, Lo CG, Cinamon G, Lesneski MJ, Xu Y, et al (2004) Lymphocyte egress from thymus and peripheral lymphoid organs is dependent on S1P receptor 1. Nature 427: 355–360. 14737169 10.1038/nature02284

[12] Allende ML, Bektas M, Lee BG, Bonifacino E, Kang J, et al (2011) Sphingosine-1-phosphate lyase deficiency produces a pro-inflammatory response while impairing neutrophil trafficking. J Biol Chem 286: 7348–7358. 21173151 10.1074/jbc.M110.171819 PMC3044991

[13] Martino A (2007) Sphingosine 1-phosphate as a novel immune regulator of dendritic cells. J Biosci 32: 1207–1212. 17954981 10.1007/s12038-007-0122-0

[14] Olivera A, Rivera J (2011) An emerging role for the lipid mediator sphingosine-1-phosphate in mast cell effector function and allergic disease. Adv Exp Med Biol 716: 123–142. 21713655 10.1007/978-1-4419-9533-9_8 PMC3214605

[15] Walzer T, Chiossone L, Chaix J, Calver A, Carozzo C, et al (2007) Natural killer cell trafficking in vivo requires a dedicated sphingosine 1-phosphate receptor. Nat Immunol 8: 1337–1344. 17965716 10.1038/ni1523

[16] Weigert A, Weis N, Brune B (2009) Regulation of macrophage function by sphingosine-1-phosphate. Immunobiology 214: 748–760. 19625101 10.1016/j.imbio.2009.06.003

[17] Kihara A, Anada Y, Igarashi Y (2006) Mouse sphingosine kinase isoforms SPHK1a and SPHK1b differ in enzymatic traits including stability, localization, modification, and oligomerization. J Biol Chem 281: 4532–4539. 16368679 10.1074/jbc.M510308200

[18] Liu H, Chakravarty D, Maceyka M, Milstien S, Spiegel S (2002) Sphingosine kinases: a novel family of lipid kinases. Prog Nucleic Acid Res Mol Biol 71: 493–511. 12102559 10.1016/s0079-6603(02)71049-0

[19] Kim RH, Takabe K, Milstien S, Spiegel S (2009) Export and functions of sphingosine-1-phosphate. Biochim Biophys Acta 1791: 692–696. 19268560 10.1016/j.bbalip.2009.02.011 PMC2763566

[20] Takabe K, Paugh SW, Milstien S, Spiegel S (2008) “Inside-out” signaling of sphingosine-1-phosphate: therapeutic targets. Pharmacol Rev 60: 181–195. 18552276 10.1124/pr.107.07113 PMC2695666

[21] Chun J, Goetzl EJ, Hla T, Igarashi Y, Lynch KR, et al (2002) International Union of Pharmacology. XXXIV. Lysophospholipid receptor nomenclature. Pharmacol Rev 54: 265–269. 12037142 10.1124/pr.54.2.265

[22] Hla T (2001) Sphingosine 1-phosphate receptors. Prostaglandins 64: 135–142. 10.1016/s0090-6980(01)00109-5 11324703

[23] Rose L, Schneider C, Stock C, Zollner TM, Docke WD (2012) Extended DNFB-induced contact hypersensitivity models display characteristics of chronic inflammatory dermatoses. Exp Dermatol 21: 25–31. 22151387 10.1111/j.1600-0625.2011.01395.x

[24] Reines I, Kietzmann M, Mischke R, Tschernig T, Luth A, et al (2009) Topical application of sphingosine-1-phosphate and FTY720 attenuate allergic contact dermatitis reaction through inhibition of dendritic cell migration. J Invest Dermatol 129: 1954–1962. 19194476 10.1038/jid.2008.454

[25] Gollmann G, Neuwirt H, Tripp CH, Mueller H, Konwalinka G, et al (2008) Sphingosine-1-phosphate receptor type-1 agonism impairs blood dendritic cell chemotaxis and skin dendritic cell migration to lymph nodes under inflammatory conditions. Int Immunol 20: 911–923. 18495625 10.1093/intimm/dxn050

[26] Baumer W, Sulzle B, Weigt H, De Vries VC, Hecht M, et al (2005) Cilomilast, tacrolimus and rapamycin modulate dendritic cell function in the elicitation phase of allergic contact dermatitis. Br J Dermatol 153: 136–144. 16029339 10.1111/j.1365-2133.2005.06745.x

[27] Hackstein H, Taner T, Logar AJ, Thomson AW (2002) Rapamycin inhibits macropinocytosis and mannose receptor-mediated endocytosis by bone marrow-derived dendritic cells. Blood 100: 1084–1087. 12130531 10.1182/blood.v100.3.1084

[28] Monti P, Mercalli A, Leone BE, Valerio DC, Allavena P, et al (2003) Rapamycin impairs antigen uptake of human dendritic cells. Transplantation 75: 137–145. 12544886 10.1097/00007890-200301150-00025

[29] Blot V, Jacquemard U, Reissig HU, Kleuser B (2009) Practical Syntheses of Sphingosine-1-Phosphate and Analogues. Synthesis-Stuttgart: 759–766.

[30] Xu S, Ariizumi K, Caceres-Dittmar G, Edelbaum D, Hashimoto K, et al (1995) Successive generation of antigen-presenting, dendritic cell lines from murine epidermis. J Immunol 154: 2697–2705. 7876542

[31] Schuhmachers G, Xu S, Bergstresser PR, Takashima A (1995) Identity and functional properties of novel skin-derived fibroblast lines (NS series) that support the growth of epidermal-derived dendritic cell lines. J Invest Dermatol 105: 225–230. 7636305 10.1111/1523-1747.ep12317512

[32] Lutz MB, Kukutsch N, Ogilvie AL, Rossner S, Koch F, et al (1999) An advanced culture method for generating large quantities of highly pure dendritic cells from mouse bone marrow. J Immunol Methods 223: 77–92. 10037236 10.1016/s0022-1759(98)00204-x

[33] Sparber F, Tripp CH, Hermann M, Romani N, Stoitzner P (2010) Langerhans cells and dermal dendritic cells capture protein antigens in the skin: possible targets for vaccination through the skin. Immunobiology 215: 770–779. 20599290 10.1016/j.imbio.2010.05.014 PMC2939980

[34] Baumer W, Tschernig T, Sulzle B, Seegers U, Luhrmann A, et al (2003) Effects of cilomilast on dendritic cell function in contact sensitivity and dendritic cell migration through skin. Eur J Pharmacol 481: 271–279. 14642795 10.1016/j.ejphar.2003.09.031

[35] Ruwisch L, Schafer-Korting M, Kleuser B (2001) An improved high-performance liquid chromatographic method for the determination of sphingosine-1-phosphate in complex biological materials. Naunyn Schmiedebergs Arch Pharmacol 363: 358–363. 11284453 10.1007/s002100000365

[36] Trombetta ES, Mellman I (2005) Cell biology of antigen processing in vitro and in vivo. Annu Rev Immunol 23: 975–1028. 15771591 10.1146/annurev.immunol.22.012703.104538

[37] Sarkar K, Kruhlak MJ, Erlandsen SL, Shaw S (2005) Selective inhibition by rottlerin of macropinocytosis in monocyte-derived dendritic cells. Immunology 116: 513–524. 16313365 10.1111/j.1365-2567.2005.02253.x PMC1802442

[38] Norbury CC (2006) Drinking a lot is good for dendritic cells. Immunology 117: 443–451. 16556257 10.1111/j.1365-2567.2006.02335.x PMC1782244

[39] Radeke HH, von Wenckstern H, Stoidtner K, Sauer B, Hammer S, et al (2005) Overlapping signaling pathways of sphingosine 1-phosphate and TGF-beta in the murine Langerhans cell line XS52. J Immunol 174: 2778–2786. 15728487 10.4049/jimmunol.174.5.2778

[40] Hong JH, Youm JK, Kwon MJ, Park BD, Lee YM, et al (2008) K6PC-5, a direct activator of sphingosine kinase 1, promotes epidermal differentiation through intracellular Ca2+ signaling. J Invest Dermatol 128: 2166–2178. 18385762 10.1038/jid.2008.66

[41] Bennett CL, Noordegraaf M, Martina CA, Clausen BE (2007) Langerhans cells are required for efficient presentation of topically applied hapten to T cells. J Immunol 179: 6830–6835. 17982073 10.4049/jimmunol.179.10.6830

[42] Bursch LS, Wang L, Igyarto B, Kissenpfennig A, Malissen B, et al (2007) Identification of a novel population of Langerin+ dendritic cells. J Exp Med 204: 3147–3156. 18086865 10.1084/jem.20071966 PMC2150989

[43] Kissenpfennig A, Henri S, Dubois B, Laplace-Builhe C, Perrin P, et al (2005) Dynamics and function of Langerhans cells in vivo: dermal dendritic cells colonize lymph node areas distinct from slower migrating Langerhans cells. Immunity 22: 643–654. 15894281 10.1016/j.immuni.2005.04.004

[44] Sullivan S, Bergstresser PR, Tigelaar RE, Streilein JW (1986) Induction and regulation of contact hypersensitivity by resident, bone marrow-derived, dendritic epidermal cells: Langerhans cells and Thy-1+ epidermal cells. J Immunol 137: 2460–2467. 2876041

[45] Bennett CL, van Rijn E, Jung S, Inaba K, Steinman RM, et al (2005) Inducible ablation of mouse Langerhans cells diminishes but fails to abrogate contact hypersensitivity. J Cell Biol 169: 569–576. 15897263 10.1083/jcb.200501071 PMC2171694

[46] Zahner SP, Kel JM, Martina CA, Brouwers-Haspels I, van Roon MA, et al (2011) Conditional deletion of TGF-betaR1 using Langerin-Cre mice results in Langerhans cell deficiency and reduced contact hypersensitivity. J Immunol 187: 5069–5076. 21998450 10.4049/jimmunol.1101880

[47] Guaguere E, Steffan J, Olivry T (2004) Cyclosporin A: a new drug in the field of canine dermatology. Vet Dermatol 15: 61–74. 15030555 10.1111/j.1365-3164.2004.00376.x

[48] Kitajima T, Caceres-Dittmar G, Tapia FJ, Jester J, Bergstresser PR, et al (1996) T cell-mediated terminal maturation of dendritic cells: loss of adhesive and phagocytotic capacities. J Immunol 157: 2340–2347. 8805631

[49] Lan YY, Tokita D, Wang Z, Wang HC, Zhan J, et al (2008) Sphingosine 1-phosphate receptor agonism impairs skin dendritic cell migration and homing to secondary lymphoid tissue: association with prolonged allograft survival. Transpl Immunol 20: 88–94. 18694829 10.1016/j.trim.2008.07.004

[50] Maeda Y, Matsuyuki H, Shimano K, Kataoka H, Sugahara K, et al (2007) Migration of CD4 T cells and dendritic cells toward sphingosine 1-phosphate (S1P) is mediated by different receptor subtypes: S1P regulates the functions of murine mature dendritic cells via S1P receptor type 3. J Immunol 178: 3437–3446. 17339438 10.4049/jimmunol.178.6.3437

[51] Ocana-Morgner C, Reichardt P, Chopin M, Braungart S, Wahren C, et al (2011) Sphingosine 1-phosphate-induced motility and endocytosis of dendritic cells is regulated by SWAP-70 through RhoA. J Immunol 186: 5345–5355. 21421853 10.4049/jimmunol.1003461

[52] Ruedl C, Koebel P, Karjalainen K (2001) In vivo-matured Langerhans cells continue to take up and process native proteins unlike in vitro-matured counterparts. J Immunol 166: 7178–7182. 11390465 10.4049/jimmunol.166.12.7178

[53] Hara-Chikuma M, Sugiyama Y, Kabashima K, Sohara E, Uchida S, et al (2012) Involvement of aquaporin-7 in the cutaneous primary immune response through modulation of antigen uptake and migration in dendritic cells. Faseb J 26: 211–218. 21968069 10.1096/fj.11-186627

[54] Kerr MC, Teasdale RD (2009) Defining macropinocytosis. Traffic 10: 364–371. 19192253 10.1111/j.1600-0854.2009.00878.x

[55] Yanagawa Y, Matsumoto M, Togashi H (2010) Enhanced dendritic cell antigen uptake via alpha2 adrenoceptor-mediated PI3K activation following brief exposure to noradrenaline. J Immunol 185: 5762–5768. 20935206 10.4049/jimmunol.1001899

[56] Schuppel M, Kurschner U, Kleuser U, Schafer-Korting M, Kleuser B (2008) Sphingosine 1-phosphate restrains insulin-mediated keratinocyte proliferation via inhibition of Akt through the S1P2 receptor subtype. J Invest Dermatol 128: 1747–1756. 18219276 10.1038/sj.jid.5701259

[57] Baumer W, Rossbach K, Mischke R, Reines I, Langbein-Detsch I, et al (2011) Decreased concentration and enhanced metabolism of sphingosine-1-phosphate in lesional skin of dogs with atopic dermatitis: disturbed sphingosine-1-phosphate homeostasis in atopic dermatitis. J Invest Dermatol 131: 266–268. 20811395 10.1038/jid.2010.252

[58] Kumar A, Saba JD (2009) Lyase to live by: sphingosine phosphate lyase as a therapeutic target. Expert Opin Ther Targets 13: 1013–1025. 19534571 10.1517/14728220903039722 PMC2774446

[59] Seo EY, Park GT, Lee KM, Kim JA, Lee JH, et al (2006) Identification of the target genes of atopic dermatitis by real-time PCR. J Invest Dermatol 126: 1187–1189. 16528358 10.1038/sj.jid.5700234

[60] Wood SH, Clements DN, Ollier WE, Nuttall T, McEwan NA, et al (2009) Gene expression in canine atopic dermatitis and correlation with clinical severity scores. J Dermatol Sci 55: 27–33. 19394200 10.1016/j.jdermsci.2009.03.005

[61] Schröder M, Richter C, Juan MH, Maltusch K, Giegold O, et al (2011) The sphingosine kinase 1 and S1P1 axis specifically counteracts LPS-induced IL-12p70 production in immune cells of the spleen. Mol Immunol 48: 1139–1148. 21435724 10.1016/j.molimm.2011.02.007

[62] van de Ven R, de Jong MC, Reurs AW, Schoonderwoerd AJ, Jansen G, et al (2006) Dendritic cells require multidrug resistance protein 1 (ABCC1) transporter activity for differentiation. J Immunol 176: 5191–5198. 16621983 10.4049/jimmunol.176.9.5191

[63] Mitra P, Oskeritzian CA, Payne SG, Beaven MA, Milstien S, et al (2006) Role of ABCC1 in export of sphingosine-1-phosphate from mast cells. Proc Natl Acad Sci U S A 103: 16394–16399. 17050692 10.1073/pnas.0603734103 PMC1637593

